# Design Scheme of New Tetragonal Heusler Compounds for Spin-Transfer Torque Applications and its Experimental Realization

**DOI:** 10.1002/adma.201201879

**Published:** 2012-09-11

**Authors:** Jürgen Winterlik, Stanislav Chadov, Arunava Gupta, Vajiheh Alijani, Teuta Gasi, Kai Filsinger, Benjamin Balke, Gerhard H Fecher, Catherine A Jenkins, Frederick Casper, Jürgen Kübler, Guo-Dong Liu, Li Gao, Stuart S P Parkin, Claudia Felser

**Affiliations:** Institut für Anorganische und Analytische Chemie, Johannes Gutenberg-UniversitätStaudinger Weg 9, 55128 Mainz, Germany; Max Planck Institute for Chemical Physics of SolidsNöthnitzer Straße 40, 01187 Dresden, Germany; Center for Materials for Information Technology and Department of Chemistry, University of AlabamaTuscaloosa, Alabama 35487, USA; Advanced Light Source Lawrence Berkeley National LaboratoryBerkeley, CA 94720, USA; Institut für Festkörperphysik Technische UniversitätKarolinenplatz 5, 64289 Darmstadt, Germany; School of Materials Sciences and Engineering, Hebei University of TechnologyTianjin 300130, China; IBM Research Division, Almaden Research CenterSan Jose, CA 95120, USA

**Keywords:** Heusler compounds, spin-transfer torques, magnetic properties, spintronics

Heusler compounds represent a remarkable class of materials with more than 1500 members. While they have been known for a long time,^1^ it is only in recent years that a wide range of their extraordinary functionalities, including half-metallic high-temperature ferri- and ferromagnets,^2^ multiferroic shape memory alloys,^3–5^ and topological insulators,^6–8^ have attracted significant attention for spintronics,^9^ energy technologies,^10^, ^11^ and magnetocaloric applications.^12^ One of the most recent advances has been the identification of the tetragonal Heusler compound Mn_3_Ga for spin-transfer torque (STT) applications.^13^, ^14^ Current development of efficient spintronic devices is focused on exploiting the STT phenomenon for non-volatile memory and logic devices. The primary advantage of STT as compared to conventional field-induced switching is the significant downscaling of the device dimensions, compatible with the needs for next-generation memory and logic devices, with reduced power consumption.^15^

The requirements, especially on the material used as the switching element in spin-transfer torque devices are quite stringent. The major challenge is to minimize the switching current and switching time while maintaining thermal stability. Additionally, growth of smooth thin films that are lattice-matched with the commonly used tunneling barrier MgO in magnetic tunnel junctions is important. Materials with high spin polarization and Curie temperature (*T*_C_), but low saturation magnetization (*M*_S_) and Gilbert damping are needed to minimize the switching current and switching speed according to the Slonczewski–Berger equation.^16^, ^17^ However, a thermal stability factor *K*_U_*V*/*k*_B_*T* ≍ 60, where *K*_U_ is the effective anisotropy and *V* the cell volume, is required to ensure non-volatility of the stored information. Therefore, in order to minimize the switching current, one wants low damping and high spin polarization. On the other hand, to minimize the switching time, one wants high damping and most importantly, a high effective anisotropy field *H*_K_, which is inversely related to the free layer moment through the thermal stability requirement, *H*_K_*M*_S_*V* = 2*K*_U_*V* ≍ 0.5 aJ. Tunability, especially of *M*_S_, in order to optimize the conflicting demands on an STT material is thus highly desirable.

A key property for realization of fast switching with low currents and high thermal stability is the perpendicular magnetocrystalline anisotropy (PMA). Initial experimental and theoretical studies of the bulk properties^14^ suggested that the tetragonally distorted Heusler alloys Mn_3–*x*_Ga (*x* = 0–1) are attractive PMA materials for STT applications. The expected strong PMA has since been realized in thin films by Miyazaki's group,^18^ and a high spin polarization confirmed by Kurt et al.^19^ More recently, an exceptionally low Gilbert damping and long-lived ultrafast spin precession with frequencies up to 280 GHz has been demonstrated in Mn_3–*x*_Ga by Mizukami et al.^20^ The primary drawback of Mn_3–*x*_Ga is the lattice mismatch with MgO, which leads to low tunnel magnetoresistance (TMR) in devices.^21^, ^22^ Furthermore, for an optimal balance between switching current, fast switching and thermal stability, the magnetic moment of Mn_3–*x*_Ga is not sufficiently low. In this communication we discuss approaches based on ab-initio theory for the design of additional tetragonal PMA Heusler compounds with tunable moment for STT applications and their experimental verification. A key advantage of Heusler materials is that many of them intrinsically exhibit high spin polarization and high *T*_C_.^23^, ^24^ Furthermore, their predictable electronic structures and magnetic properties allow for tunability with suitable substitution. Tetragonal Heusler compounds could thus satisfy the unique requirements of materials for STT-based memory and logic devices, and also for spin torque oscillators (STO) currently being investigated for telecommunications.

Electronic instabilities corresponding to the band Jahn–Teller type, which causes large tetragonal distortions of the cubic Heusler structure, was reported in Rh_2_-based Heusler compounds by Suits in 1976.^25^ This type of distortion is now readily predictable based on band structure calculations. The underlying idea is that the structural instability of the cubic phase is typically indicated by van Hove singularities^26^ in proximity of the Fermi energy (*E*_F_) resulting in high peaks of the density of states (DOS). These singularities can be straightforwardly identified by ab-initio band structure calculations (see the Supporting Information). If all reasonable electronic relaxation mechanisms including magnetism cannot eliminate the singularity, the only way to escape from this type of instability is by undergoing a structural distortion, thereby reducing the DOS at *E*_F_. To take it to the next level, this characteristic feature can be utilized for the design of tetragonal Heusler compounds by tuning the chemical composition of a compound with suitable substitution to shift the van Hove singularity close to *E*_F_ in order to force the distortion. Following this ansatz we have synthesized a number of stoichiometric tetragonal Heusler compounds and corresponding alloys, and expect that many other alloys can be identified following the proposed design scheme.

**Figure 1:**
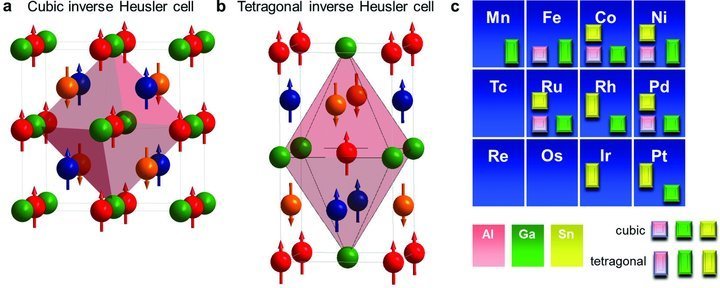
a,b) Cubic and tetragonal cells of Mn_2_YZ Heusler compounds. The arrows denote the orientation of the corresponding magnetic moments of the atoms. The red and orange balls represent Mn atoms, whereas the blue and green ones represent the transition and the main group metals, respectively. The three different sublattices can be readily visualized. c) The structure of Heusler compositions calculated, synthesized, and analyzed with Y = Group 7–10 elements from the periodic table and Z = Al, Ga, and Sn. The structures were determined using X-ray powder diffraction and Rietveld analyses (see Supporting Information). The preferred structure chosen by a specific composition depends strongly on the electronic structure of the compound, which is related to van Hove singularity.

With respect to the requirements of STT materials, the most promising systems are those based on Mn_2_ combined with transition metals that are more electronegative than Mn. For these combinations, the inverse cubic Heusler structure with three distinct magnetic sublattices is formed, as shown in **Figure** **1**a. The corresponding tetragonally distorted inverse Heusler structure is shown in Figure 1b. Because of the interatomic distances, the Mn atoms in the different sublattice couple antiferromagnetically ensuring the desired low effective magnetic moment. In Heusler compounds the octahedrally coordinated Mn atoms typically exhibit highly localized *d*-bands close to *E*_F_ and are thus very susceptible to band Jahn–Teller distortions (similar to *d*^4^ Mn^3+^ ions in an octahedral crystal field). The typical *c/a* value for tetragonal Heusler compounds is often close to 1.3, but can vary in the range 0.95 < *c/a* > 1.43. What remains is to evaluate the relative stability of the material for different distortions. The derived energy profiles allow for distinguishing between a stable tetragonal distortion (e.g., Mn_3_Ga), a stable cubic structure (e.g., Mn_2_CoGa), and a shape memory system (e.g., Mn_2_NiGa) (the corresponding results of the first-principles calculations are shown in Figure S1 in the Supporting Information). Typical profiles of stable cubic compounds exhibit the total energy minimum at *c/a* = 1, those of tetragonal compounds at *c/a* ≠ 1. Shape memory compounds exhibit two distinct minima (cubic and tetragonal structures) separated by a small energy barrier. Mn_2.5_Co_0.5_Ga happens to be a system which lies exactly at the borderline between the stable and unstable cubic structures. Figure 1c provides an overview of several cubic and tetragonal Mn_2_YZ Heusler compounds with Z = Al, Ga, and Sn. Most of these materials have not been reported earlier and we have synthesized and characterized them experimentally for the first time following the structure-property relations for Mn_2_YZ. The current literature reports only Mn_3_Ga, Mn_2_CoGa, Mn_2_CoSn, Mn_2_NiGa, Mn_2_NiSn, and Mn_2_RuGa.^13^, ^27–30^ Three trends are apparent from Figure 1c: Mn_2_YAl compounds only form cubic structures. Mn_2_YGa compounds with the exception of Y = Co tend to form tetragonal structures with *3d* elements at the Y position. In contrast, the *4d* and *5d* elements form only cubic Mn_2_YGa. The opposite situation occurs in Mn_2_YSn. Several tetragonal compounds with *4d* and *5d* elements are formed, while only cubic (and hexagonal, whose details are not provided here) phases are stabilized with *3d* elements. Based on band structure calculations, all the tetragonal compounds exhibit a small energy difference between the van Hove singularity and *E*_F_.

**Figure 2:**
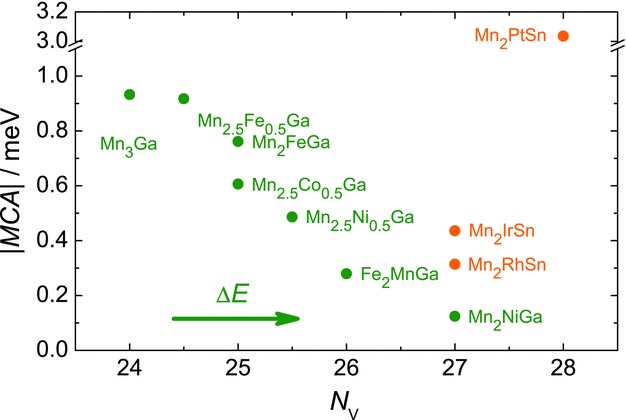
The moduli of the magnetocrystalline anisotropy (|MCA|) calculated for several tetragonal Heusler compounds. The |MCA| of Mn_2_YGa compounds with Y = *3d* transition metal increase when Δ*E* becomes smaller (see text). The smallest |MCA| is consequently found for the shape-memory compound Mn_2_NiGa and the highest values are found for coincidence of the van Hove singularity with *E*_F_. The |MCA| increases with increasing SOC as well (see Mn_2_YSn). A higher SOC is at the expense of higher Gilbert damping, so balancing is important.

It is important to emphasize that while tetragonal distortion is a necessary condition, it does not guarantee perpendicular magnetization in thin films as reported recently for the related tetragonal Heusler compounds Rh_2_YZ.^31^ This is because the magnetocrystalline anisotropy (MCA) tends to oscillate as a function of *c/a*. **Figure** **2** shows the calculated MCA energies of various tetragonal Heusler compounds. The MCA of the Mn_2_-based compounds are found to follow some simple trends. First, their MCA strongly depends on the number of valence electrons (*N*_V_), which is directly related to Δ*E*, the difference in energy between the van Hove singularity and *E*_F_; second, the MCA scales with spin-orbit coupling (SOC) – increasing in going from Y = *3d* (Ni) to *4d* (Rh) to *5d* (Ir). The highest MCA is found for Mn_2_PtSn (3.04 meV) but its low *T*_C_ (374 K) constitutes a drawback. On the other hand, the lowest MCA values are observed in the known shape memory systems Mn_2_NiGa and Fe_2_MnGa. Mn_3_Ga with a calculated MCA of about 1 meV, which is in excellent agreement with experimental observation,^14^ is competitive with FePt (MCA close to 3 meV)^32^ that is also a potential candidate for STT applications. As the anisotropy fields of both materials are similar, the difference in MCA is due to the lower magnetic moment of Mn_3_Ga, which is actually desirable for faster switching of STT devices. Mn_3_Ga also exhibits a substantially lower (one order of magnitude) Gilbert damping constant and much higher TMR as compared to FePt.^19^, ^20^ Mn_2.7_Co_0.3_Ga is even more attractive than Mn_3_Ga since its magnetic moment is about half the size but with sufficiently large MCA. This is evidently the point where tuning the relevant properties by alloying comes into play, and the important message is that Heusler alloys exhibit stable tetragonal structures over a wide range of compositions.

**Figure 3:**
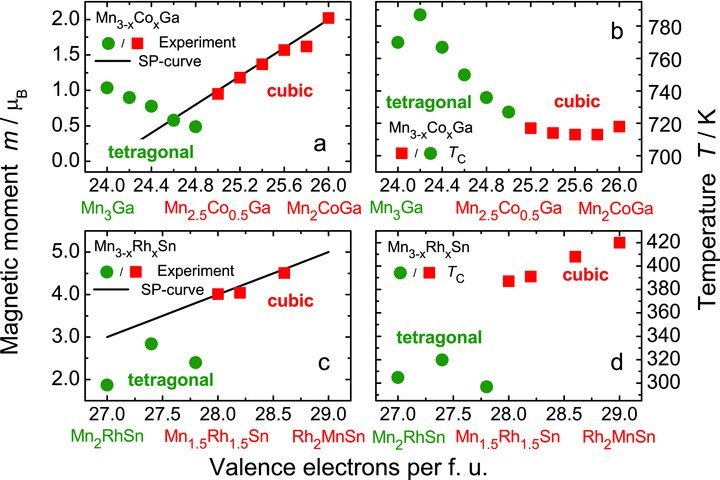
a,c) Saturation magnetic moments of Mn_3–*x*_Co_*x*_Ga^28^ and Mn_3–*x*_Rh_*x*_Sn alloys measured at *T* = 5 K and compared with the Slater–Pauling values. Tetragonal and cubic compounds are represented by the circles and squares, respectively. b,d) The corresponding *T*_C_ of the alloys. The composition Mn_1.4_Rh_1.6_Sn contains an unidentified impurity causing a large error in the magnetic moment.

Starting with the stoichiometric Mn_3_Ga compound we can explore the complete phase diagram of Mn_3–*x*_Y_*x*_Z. As illustrations, we consider the detailed experimental characterization of the systems Mn_3–*x*_Co_*x*_Ga and Mn_3–*x*_Rh_*x*_Sn. The measured magnetic moment and *T*_C_ for different compositions in the two systems are plotted in **Figure** **3**. Partially substituting Mn by Co leads to the Mn_3–*x*_Co_*x*_Ga system, with the tetragonal structure being stable for Co concentrations as high as *x* = 0.4. All these tetragonal alloys are magnetic and exhibit high MCA, similar to Mn_3–*x*_Ga, but with even lower *M*_S_. The alloy Mn_2.5_Co_0.5_Ga is a phase mixture consisting of both tetragonal and cubic structures, while the Co-rich alloys are cubic and magnetically soft. While the tetragonal alloys exhibit features attractive for STT applications (high *T*_C_, strong PMA, high spin polarization, low *M*_S_), the cubic systems represent a large class of 100% spin polarized half-metallic Heusler materials that robustly follow the Slater–Pauling rule similar to the Co_2_YZ compounds.^33^ Note that the tetragonal Mn_3–*x*_Co_*x*_Ga alloys are also highly spin-polarized due to a pseudo-gap in one spin channel. Thus, tuning the spin polarization and the magnetic moment offers the opportunity for systematic tailoring the magnetic properties. The magnetic interactions in Mn_3–*x*_Co_*x*_Ga correspond to the arrangements shown in Figure 1. The Co atoms are all tetrahedrally coordinated, while the Mn atoms are located in both tetrahedral and octahedral environments. Being next neighbours, they carry opposing spins. Even though the alloys Mn_2.6_Co_0.4_Ga and Mn_2.7_Co_0.3_Ga are superior to Mn_3_Ga as STT materials because of their lower moment, they exhibit large lattice mismatch with MgO (*a* = 4.212 Å, Mn_2.6_Co_0.4_Ga *a* = 3.892 Å, Mn_2.7_Co_0.3_Ga *a* = 3.874 Å). The mismatch can be reduced by introducing larger atoms such as Sn. In comparison, Mn_2_RhSn is a tetragonal Heusler compound with moderate MCA and a lattice mismatch of only 1.8% with MgO (Mn_2_RhSn *a* = 4.294 Å). The tetragonal phase of Mn_3–*x*_Rh_*x*_Sn has been experimentally determined to be stable up to *x* = 0.4, as shown in Figure 3c. The alloys for *x* = 0.5 > *x* > 1 are cubic and follow the Slater–Pauling rule similar to Mn_3–*x*_Co_*x*_Ga. The drawback of the Mn_3–*x*_Rh_*x*_Sn compounds is their low *T*_C_, impeding application at convenient operating temperatures. However, the *T*_C_ can be increased for instance by doping with Co. Another option is to increase the content of Mn, since the tetragonal structure is expected to be stable for the Mn-rich compositions. The experimental verification of these ideas is our next task.

In summary, our results unambiguously demonstrate that the phase space of tetragonal Heusler compounds is much larger than only Mn_3–*x*_Ga, and that the important STT parameters can be tailored by adjustments of the composition. A significant amount of work remains, but following the path outlined here it should be possible to design a wide range of Heusler STT materials with PMA that fulfill all the requirements: complete tunability of the magnetic moment, the lattice parameters, MCA, and SOC, which is necessary for fulfilling some of the conflicting requirements for low switching current, fast switching and thermal stability. A number of the tetragonal Heusler compounds offer high spin polarization, high *T*_C_, and low Gilbert damping due to moderate SOC of the *3d* and *4p* elements as compared to other anisotropic magnetic alloys such as FePt. Another potential advantage of Mn_3–*x*_-based Heusler alloys with respect to the fabrication of magnetic tunnel junctions is that with three partially antiferromagnetically coupled sublattices they inherently offer all the prerequisites currently realized using complex synthetic ferrimagnet structures consisting of three layers in one device. Thus, two of the layers can be eliminated when substituted by a highly spin-polarized Heusler ferrimagnet.
