# Supplementary material for: Design Scheme of New Tetragonal Heusler Compounds for Spin-Transfer Torque Applications and its Experimental Realization
Source: Adv Mater. 2012 Sep 11;24(47):6283–7. doi: 10.1002/adma.201201879 (PMC3546386; doi:10.1002/adma.201201879)
Supplement: Supplementary file 1 [file adma0024-6283-SD1.pdf]

Copyright WILEY-VCH Verlag GmbH & Co. KGaA, 69469 Weinheim, Germany, 2012.

ADVANCED MATERIALS

Supporting Information

for *Adv. Mater.*, DOI: 10.1002/adma.201201879

Design Scheme of New Tetragonal Heusler Compounds for Spin-Transfer Torque Applications and its Experimental Realization

*Jürgen Winterlik, Stanislav Chadov, Arunava Gupta, Vajiheh Alijani, Teuta Gasi, Kai Filsinger, Benjamin Balke, Gerhard H. Fecher, Catherine A. Jenkins, Frederick Casper, Jürgen Kübler, Guo-Dong Liu, Li Gao, Stuart S. P. Parkin, and Claudia Felser**

Supporting Information

For Adv. Mater. DOI: 10.1002/adma.201201879

Design Scheme of New Tetragonal Heusler Compounds for Spin-Transfer Torque Applications and its Experimental Realization

Jürgen Winterlik, Stanislav Chadov, Arunava Gupta, Vajiheh Alijani, Teuta Gasi, Kai Filsinger, Benjamin Balke, Gerhard H. Fecher, Catherine A. Jenkins, Frederick Casper, Jürgen Kübler, Guo-dong Liu, Li Gao, Stuart S. P. Parkin, and Claudia Felser*

Supporting Information

Design Scheme of New Tetragonal Heusler Compounds for Spin-Transfer Torque Applications and its Experimental Realization

Jürgen Winterlik, Stanislav Chadov, Arunava Gupta, Vajiheh Alijani, Teuta Gasi, Kai Filsinger, Benjamin Balke, Gerhard H. Fecher, Catherine A. Jenkins, Frederick Casper, Jürgen Kübler, Guo-dong Liu, Li Gao, Stuart S. P. Parkin, and Claudia Felser*

Calculation details

All calculations were performed by means of the first-principles band-structure fully-relativistic version (A. Perlov, A. Yaresko, V. Antonov. Spin-polarized relativistic linear muffin-tin orbitals package for electronic structure calculations, PY-LMTO, unpublished) of the standard linearized muffin-tin orbitals approach. The exchange–correlation part of the effective potential was treated by using the Vosko–Wilk–Nusair parameterization[1] of the local-density approximation. The relative stability of the unit cell geometry was determined by calculating the total energy as a function of the *c/a* ratio at constant volume (see Figure S1).

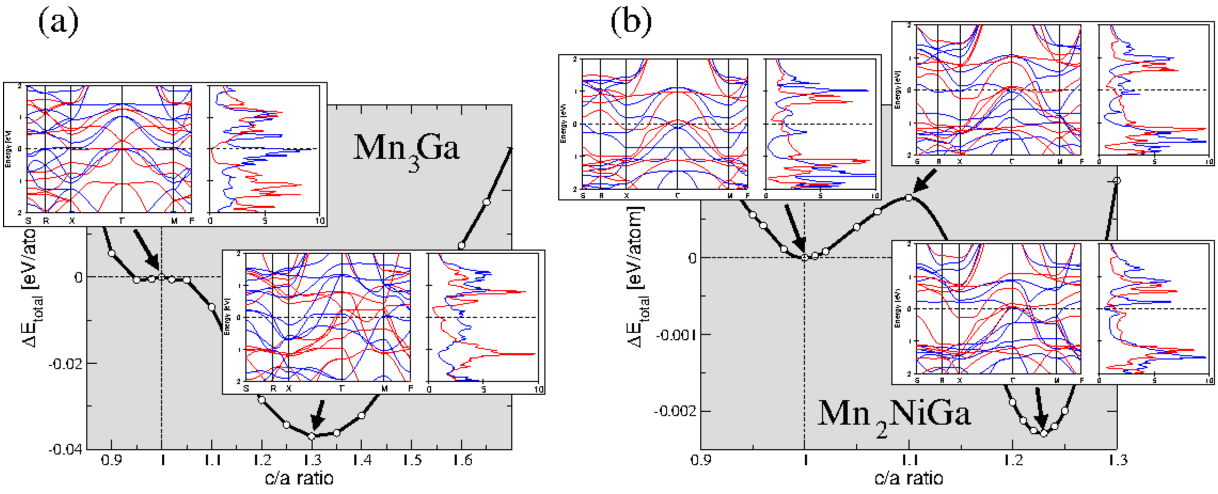

Figure S1: Determination of the relative phase stability. The total energy difference between cubic and tetragonally distorted phases calculated as a function of *c/a*: $\Delta E_{total} = E_{\text{total}}(c/a) - E_{\text{total}}(c/a = 1)$. The red and blue points mark the non-stable and stable configurations, respectively. a), The Mn_3Ga system. The insets show the corresponding spin-resolved band

structures and densities of states for the cubic (c/a = 1) and distorted (c/a = 1.3) cases. The bold curves correspond to the spin channel, which exhibits the van Hove singularity at the Fermi energy in the cubic case. The latter is obvious in the band structure (at R, X, Γ and M symmetric points in the Brillouin zone, symmetry group 119) and leads to a high peak of DOS at E_F in this spin channel. The tetragonal distortion lifts this degeneracy by moving the states away from the Fermi energy, which reduces the DOS peaks and stabilizes the system at c/a=1.3. b), The shape-memory alloy Mn_2NiGa. The system exhibits two stable states at c/a=1 and c/a=1.23. In the non-stable configuration (c/a=1.1) the singularity in R-X k-direction leads to a sharp DOS peak at E_F. It is evident from the magnitude of energy difference between cubic and tetragonal phases why Mn_3Ga is stable in the tetragonal phase while Mn_2NiGa is a shape memory compound.

The latter condition follows from both experimental and theoretical estimations that the changes in volume are related with a significantly larger energy scale when comparing the changes in geometric. The volume was thus determined only once by optimizing the cubic structure for each system. The magnetic anisotropy was calculated according to its definition, as the total energy difference between states with magnetization direction along the tetragonal strain (001) and in the perpendicular direction (010). Figure S2 shows the effect of the tetragonal distortion on the band structure and the corresponding symmetries.

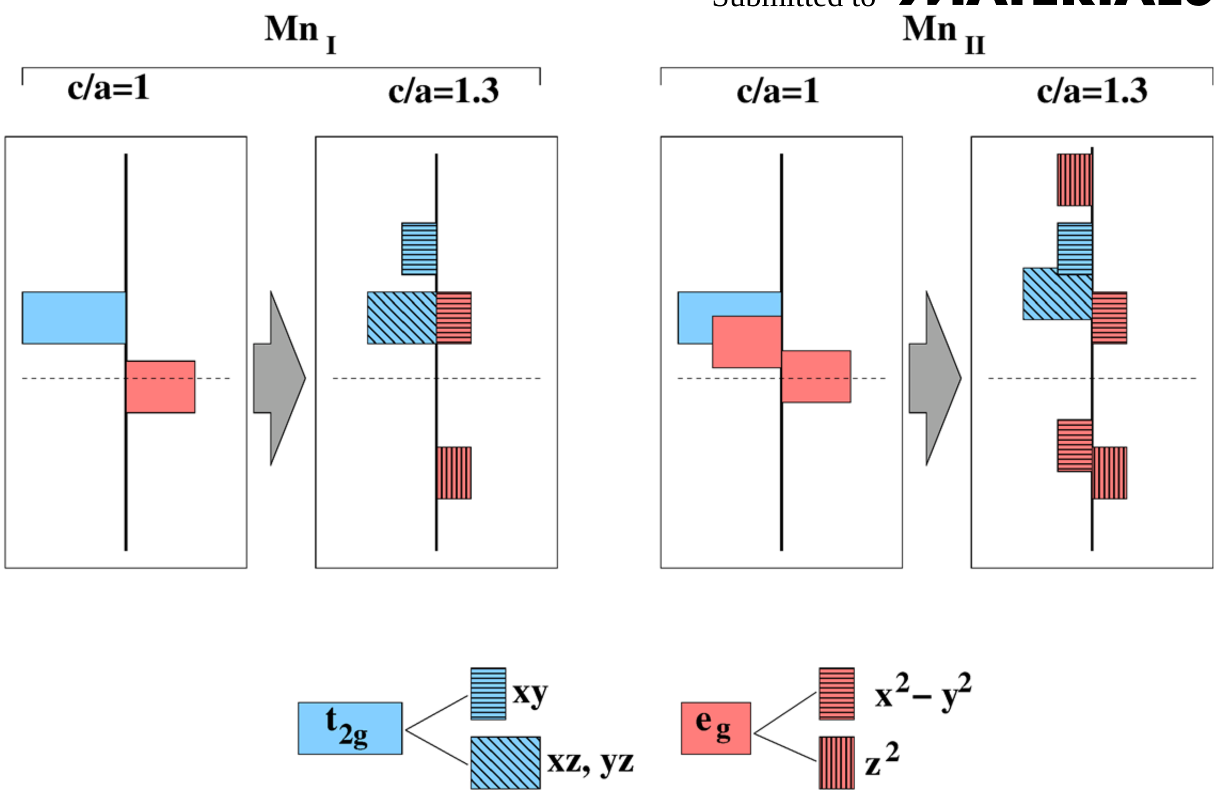

Figure S2: Effect of the tetragonal strain on the orbital structure. The example corresponds to the Mn_3Ga system. The horizontal bars mark the *d*-energy levels of the octahedrally- (Mn-I) and tetrahedrally-coordinated (Mn-II) atoms calculated at the Γ point of the Brillouin zone. Left or right orientation of a bar distinguishes the “up” or “down” spin channels, respectively (also indicated by black arrows). The size of a bar corresponds to the relative spectral weight of the specific symmetry distinguished by color. In the cubic crystal (c/a=1) the *d*-orbitals group in twofold e_g (z^2, x^2-y^2) and threefold t_{2g} (*xy*, *xz*, *yz*) representations of the octahedron symmetry group, marked by violet and light-green, respectively. By undergoing tetragonal strain along the *z*-axis (c/a=1.3), the out-of-plane orbitals split from the in-plane ones. In particular, the e_g-orbitals at E_F, which carry the van Hove singularity in the “down” spin channel, split into x^2-y^2 (blue) and z^2 (violet) above and below the Fermi energy, thereby reducing the corresponding DOS peak.

Synthesis of tetragonal Mn_2YZ Heusler compounds

Mn_2YZ bulk samples were prepared by repeated arc melting of stoichiometric amounts of high purity elements in argon atmosphere. To avoid oxygen contamination a Ti sponge was used as an oxygen absorber. The samples were melted three times and turned over in-between to guarantee for sufficient homogeneity. The resulting polycrystalline ingots were subsequently annealed in evacuated quartz tubes for two weeks at temperatures in the range of 573 - 1273 K, depending on the composition of the alloys. Flat disks were cut from the ingots and polished for further investigations of the bulk samples.

Characterization of tetragonal Mn_2YZ Heusler compounds

For x-ray powder diffraction (XRD), a part of the sample was ground to a fine powder. The XRD measurements were carried out at room temperature using Seifert XRD 3003 PTS and Bruker D8 diffractometers equipped with a Cu K_α and Mo $K_{\alpha1/\alpha2}$ x-ray tubes. Rietveld refinements of the experimental data were carried out using the TOPAS ACADEMIC software package[2]. The magnetic properties were investigated using a superconducting quantum interference device (SQUID, Quantum Design MPMS-XL-5). Small, nearly spherical sample pieces of approximately 20 mg were used for these measurements. Figures S3-S6 exemplarily show the diffraction patterns and Rietveld refinements for several tetragonal Heusler compounds synthesized and analyzed following our proposed design scheme. The corresponding lattice parameters and standard deviations are shown in Table S1 together with calculated values.

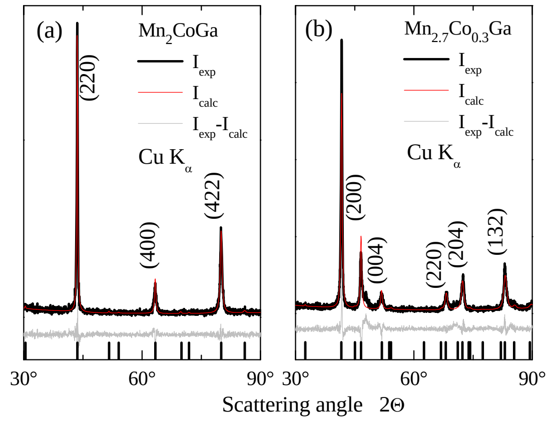

Figure S3: XRD patterns and Rietveld refinements of Mn_2CoGa and $Mn_{2.7}Co_{0.3}Ga$.

(a) displays a cubic Heusler pattern while (b) the alloy with larger Mn content is tetragonal.

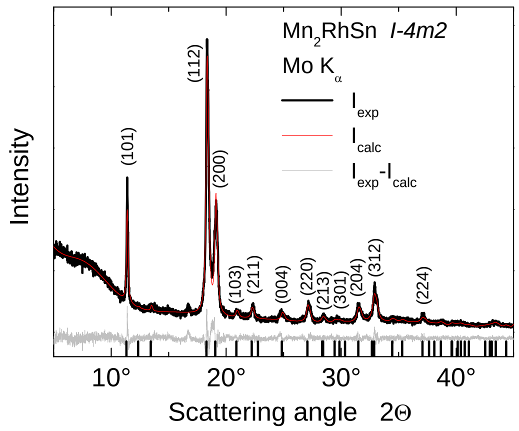

Figure S4: XRD pattern and Rietveld refinements of tetragonal Mn_2RhSn.

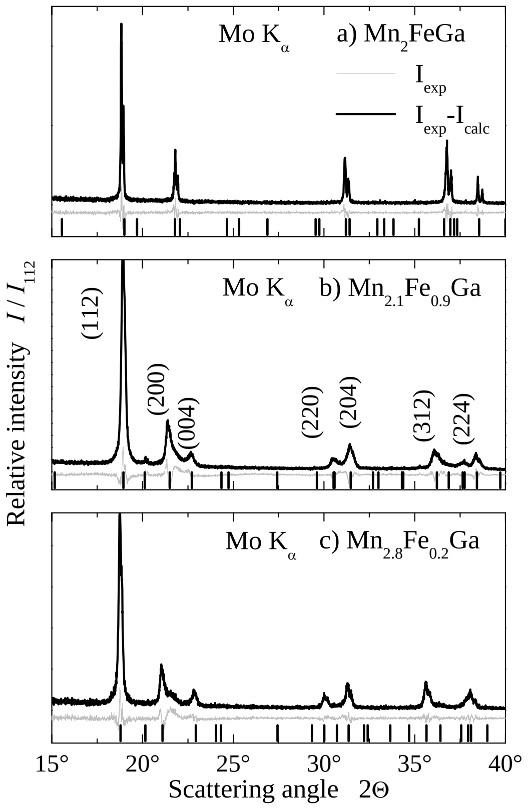

Figure S5: XRD patterns and Rietveld refinements of Mn_2FeGa, $Mn_{2.1}Fe_{0.9}Ga$ and $Mn_{2.8}Fe_{0.2}Ga$.

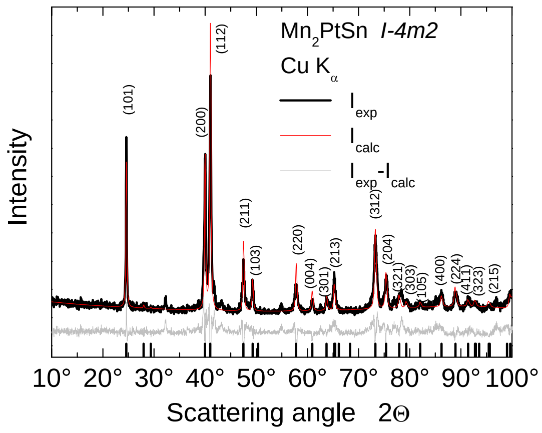

Figure S6: XRD pattern and Rietveld refinements of tetragonal Mn_2PtSn.

Table S1: Lattice parameters and errors of the diffraction data shown in Figures S3-S6. Note that *c/a* represents the value divided by the square root of 2 in order to account for the differences between the face-centered cubic and body-centered tetragonal structures. c/a_{calc} are the calculated ratii.

Alloy	*a*	*Δ a*	*c*	*Δ c*	*c/a*	c/a_{calc}
Unit	[Å]	[Å]	[Å]	[Å]	-	-
Mn_2CoGa	5.869	0.001	-	-	-	-
$Mn_{2.7}Co_{0.3}Ga$	3.874	0.003	7.024	0.005	1.28	-
$Mn_{2.5}Co_{0.5}Ga$	3.798	0.005	7.027	0.009	1.31	1.35
Mn_2RhSn	4.295	0.005	6.606	0.007	1.09	1.10
Mn_2FeGa	3.733	0.002	7.572	0.003	1.43	1.40
$Mn_{2.1}Fe_{0.9}Ga$	3.824	0.005	7.233	0.008	1.34	-
$Mn_{2.8}Fe_{0.2}Ga$	3.874	0.005	7.136	0.008	1.30	-
Mn_2PtSn	4.509	0.003	6.077	0.004	0.95	0.83
Mn_3Ga	3.909	0.002	7.098	0.003	1.28	1.30
Mn_2Ga	3.905	0.002	7.193	0.003	1.30	1.30
Mn_2NiGa	3.926	0.003	6.696	0.005	1.21	1.23

Table S2 summarizes determined structural and magnetic properties for certain tetragonal Heusler compounds and alloys.

Table S2: Experimentally determined properties of tetragonal Mn_2-based Heusler compounds. N_V is the number of valence electrons per formula unit, m_S are the saturation magnetic moments (m_{calc} are the calculated values), T_C the Curie temperatures, H_c the coercivities, and $|MAE|$ the calculated Magnetocrystalline anisotropies per formula unit.

Alloy	N_V	m_S	m_{calc}	T_C	H_c	$\|MAE\|$
Unit	-	[μ_B]	[μ_B]	[K]	[kAm^{-1}]	[meV]
Mn_3Ga	24	1.04	0.95	779	453	0.93
Mn_2FeGa	25	0.96	0.88	702	56.9	0.76
$Mn_{2.7}Co_{0.3}Ga$	24.6	0.58	-	750	282	-
Mn_2Ga	17	1.41	1.45	723	102	0.82
Mn_2RhSn	27	1.87	1.69	305	55.2	0.31
Mn_2PtSn	28	3.69	3.96	374	78.8	3.04
Mn_2NiGa	27	1.69	1.03	585	32.6	0.13

References

[1] S. H. Vosko, L. Wilk, M. Nusair, *Can. J. Phys.* **1980**, *58*, 1200–1211.

[2] A. Coelho, TOPAS ACADEMIC, version 4.1 **2007**.
